# Apoptotic and stress signaling markers are augmented in preeclamptic placenta and umbilical cord

**DOI:** 10.1016/j.bbacli.2016.05.003

**Published:** 2016-05-25

**Authors:** Syeda H. Afroze, Ram R. Kalagiri, Michelle Reyes, Jacqueline D. Zimmerman, Madhava R. Beeram, Nathan Drever, David C. Zawieja, Thomas J. Kuehl, Mohammad N. Uddin

**Affiliations:** a Department of Medical Physiology, Texas A&M Health Science Center College of Medicine, Temple, TX, USA; b Department of Pediatrics, Baylor Scott & White Health, Texas A&M Health Science Center College of Medicine, Temple, TX, USA; c Department of Obstetrics and Gynecology, Baylor Scott & White Health, Texas A&M Health Science Center College of Medicine, Temple, TX, USA; d Department of Internal Medicine, Baylor Scott & White Health, Texas A&M Health Science Center College of Medicine, Temple, TX, USA

**Keywords:** Apoptotic, Stress, Placenta, Pregnancy, Preeclampsia

## Abstract

**Objective:**

Preeclampsia (preE) has a significant link to alterations of placental function leading to stress and apoptotic signaling, which pass the placental barrier and leave persistent defect in the circulation of the offspring. We assessed apoptotic signaling in placentas and umbilical cords from patients with and without preE.

**Methods:**

We collected placental and cord tissues from 27 normal pregnant (NP) women and 20 preE consenting patients after delivery in an IRB approved prospective study. p38 mitogen-activated protein kinase (p38 MAPK) phosphorylation, pro-apoptotic Bcl-2-associated X (Bax), anti-apoptotic Bcl-2, caspase-9, and pro-inflammatory cyclooxygenase-2 (Cox-2) were evaluated by western blot and immunohistochemistry. Comparisons were performed using Student's *t*-test.

**Results:**

p38 phosphorylation (Placenta: 1.5 fold, Cord: 1.7 fold), ratio of Bax/Bcl-2 (Placenta: 1.7 fold, Cord: 2.2 fold), caspase-9 (Placenta: 1.5 fold, Cord: 1.8 fold) and Cox-2 (Placenta: 2.5 fold, Cord: 2.3 fold) were up-regulated (p < 0.05) in preE compared to NP patients. Average hospital stays for preE babies were longer than NP babies. No complications were reported for NP babies; however, all of preE babies had multiple complications.

**Conclusions:**

Apoptotic and stress signaling are augmented in preE placenta and cord tissue that alter the intrauterine environment and activates the detrimental signaling that is transported to fetus.

## 1 Introduction

Preeclampsia (preE), is a clinical syndrome characterized by a systolic blood pressure (BP) of ≥ 140 mm Hg or a diastolic BP of ≥ 90 mm Hg accompanied by proteinuria of ≥ 0.3 g in a 24-hour urine sample that begins after 20 weeks gestation [1], [2], [3], [4]. Its prevalence rate is 3 to 8% worldwide and it is one of the leading causes of maternal and fetal morbidity and mortality [1], [2], [3], [4]. The mechanism of preE is extensively being studied; however the reason why preE occurs is elusive. Several theories point to abnormal placentation caused by hypoxic insults and oxidative stress leading to placental apoptosis is being pursued [1], [2], [3], [4]. The placenta in turn releases vasculotoxic substances which passes the placental barrier and goes to the circulation of the offspring that may predispose to a pathological response to the fetus, upon birth or later in life [1], [2], [3], [4].

Theories proposed to cause preeclampsia include angiogenic imbalance [2], [5], [6], autoantibodies to angiotensin type 1 (AT_1_) receptor [7], [8], cardiotonic steroids [9], [10], [11], genetic predisposition [3] and immunological factors [12]. The angiogenic imbalance is the effect of vascular endothelial growth factor (VEGF) on the proliferation and survival of endothelial cells. It has a vasodilator effect on the systemic vessels that increases vascular permeability [2], [3], [5], [12]. However, it was found that there is an increased sensitivity to elevated levels of circulating angiotensin receptor AT_1_ activating antibody that may lead to preE [7], [8]. The cardiotonic steroids particularly Marinobufagenin (MBG) from the bufodienolide group has the ability to inhibit the Na^+^/K^+^ ATPase, increase plasma MBG promotes volume expansion and hypertension [3], [9], [10], [11]. MBG has also been shown to alter the proliferation and migration of specialized cytotrophoblasts (CTBs) in the placenta thereby decreases the perfusion of the feto-maternal unit resulting in oxidative stress and endothelial dysfunction [9], [10], [11], [13]. MBG plays a major role in the pathogenesis of preE including activation of apoptotic signaling and alteration of endothelial cell growth [3], [11], [14].

preE can occur through two connected pathways placental trophoblast dysfunction and endothelial dysfunction within the maternal systemic vasculature. The formation of various toxic compounds within the placenta such as vasoconstricting agents and altered cytokines can cause greater oxidative stress leading to endothelial dysfunction [15]. This refers to the fact that several endothelial cells did not show the proper response to specific stimuli in preE women. One study by Gant et al. found vascular resistance produced in response to increases in levels of angiotensin II was lost in preE patients [16], [17]. Possible culprit factors for endothelial dysfunction include Platelet-activating factor and P-selectin, which when unregulated favored increased platelet activity and endothelial retraction [18], [19]. Once a preE pregnancy is terminated however, disturbances in maternal circulation dissolve rapidly due to elimination of these placental factors [19]. Indeed, when endothelial dysfunction is combined with pre-existing conditions such as vascular, renal, and metabolic diseases and other genetic factors, there is a much greater risk for developing preE. While placental pathophysiology is not the primary pathway for developing preE, it is an important contributor in the development of the disorder during pregnancy.

In this prospective study, we assessed the apoptotic and stress signaling proteins in the placenta and umbilical cord of normal pregnant and preE patients The outcomes of the pregnancy were also been followed. The purpose of the study is to correlate the presence of stress and apoptotic signaling markers in the placenta and umbilical cord and their relationship to the outcome in the offspring.

## 2 Methods and materials

### 2.1 Human subjects

In this study, we recruited 20 pregnant women who present with preeclampsia defined as BP ≥ 140/90 with proteinuria of > 300 mg of protein in a 24  hours urine sample and 27 pregnant women with uncomplicated pregnancy as control from the department of Obstetrics and Gynecology at Baylor Scott & White Hospital in Temple Texas. The hospital's Institutional Review Board approved the study and informed consent was granted by the subjects. The clinical status and assessments at the time of admission for maternal symptoms and maternal age were taken. The infants' weight, length, gestational age, length of hospitalization and associated morbidities were recorded. Samples of placenta and umbilical cord were collected from the recruited subjects after delivery.

The p38 MAPK phosphorylation was evaluated by Western blot. Apoptotic markers; Bcl-2 associated X protein (Bax), pro-apoptotic Bcl-2 protein, caspase-9 and pro-inflammatory protein cyclooxygenase-2 (Cox-2) expression were assayed both by Western blot and immunohistochemistry.

### 2.2 Western blot analysis for p38 MAPK, Bax, Bcl-2, caspase-9 and Cox-2 proteins

Placenta and umbilical cord were homogenized with a cell lysis buffer (Cell Signaling Technology) containing 0.1 M Tris at pH 7.4, 50 M NaCl, 0.5 M EDTA at pH 8, Igepal and water and protease inhibitor cocktail. Protein concentrations were determined by BCA reagent (Pierce, Rockford, IL). An equal amount of protein in each sample was separated using NuPage Novex 4–12% Bis-Tris gels (Invitrogen) and transferred to nitrocellulose membranes. Membranes were blocked in 5% milk probed with anti-p38 MAPK, Bax, Bcl-2, caspase-9 and Cox-2 antibodies. After incubation with the corresponding secondary antibody, proteins were visualized with chemiluminescence detection system (Pierce). The intensity of the bands was determined using ImageQuant LAS 4000 (GE Healthcare Life Sciences). The expression of p38 MAPK, Bax, Bcl-2, caspase-9 and Cox-2 was quantified by densitometry analysis using Image J software where the target protein is normalized to a structural protein (β-actin) to control between groups and ensures correction for the amount of total protein on the membrane. The phospho-p38 was normalized to total p38.

### 2.3 Immunohistochemistry (IHC) of placenta and umbilical cord samples from preE and NP patients

Placenta and umbilical cord samples were frozen in Optimal Cutting Temperature (OCT) compound and cut on the Cryostat as 20 micron thick slices. Tissue slices were put on positively charged slides. Slides were incubated at 37 °C for 15 min and washed in PBS (phosphate buffered saline) for 5 min. Slides were places in 0.01% hydrogen peroxide for 20 min and washed in PBS for 5 min. A hydrophobic pen was used to circle tissue sections and 5% goat serum was added to the circled tissue sections for 2 h. Slides were placed in a humidified box and the Anti-Bax, Bcl-2, caspase-9 and Cox-2 antibodies (Abcam) were added in 1% goat serum. Negative control samples were placed in the humidified box without the antibody and only 1% goat serum. The humidified box was placed in the refrigerator overnight. Slides were washed in PBS three times for 30 min. The secondary antibody was added in 1% goat serum to slides for 2 h. Then slides were again washed in PBS three times for 30 min. Diaminobenzidine was added to the slides for less than 30 s then washed in water. Slides were then dipped 10 times in 50% alcohol, 70% alcohol, 90% alcohol, 95% alcohol, and xylene consecutively. Finally, dibutyl phthalate xylene mountant was added to the slides and a coverslip was placed over the tissue.

### 2.4 Statistical methods

Heterogeneity of variance of patient characteristics and assay values were examined with Levene's test. For those measurements with heterogeneous variance, Mann-Whitney *U* tests were used for comparisons of the two patient groups. Otherwise, Student's *t*-tests were used to compare groups. Data from the NP patients were compared to preE patients using Student's *t*-test. A p-value of less than 0.05 was considered significant.

## 3 Results

### 3.1 Human data

There was no significant difference between the normal pregnant and preE patients in terms of maternal age, maternal height and mean gestational age at birth. As expected, pregnant patients with symptoms of preE differed from those with normal pregnancies in variables related to these symptoms. The mean systolic BP for the preE patients (166 ± 11 mm Hg) differed from the normal patients (122 ± 10 mm Hg) respectively (p < 0.0001). The mean diastolic BP differed were 93 ± 10 and 74 ± 9 mm Hg, respectively (p 0.0001). The mean urinary protein levels differed was 1974 ± 1149 mg/24 h in the preE group and 149.0 ± 56.6 mg/24 h in the NP group (p = 0.0001). We did not find any difference in body mass index (BMI) between NP (30.6 ± 6.9 (27)) and preE women (34.2 ± 7.8 (20)), p = 0.095. Patients with NP were about 4 weeks further along in pregnancy than those with preE pregnancies, but the range of gestations for both groups was 28 to 39 weeks of gestation (Table 1).

**Fig. 1:**
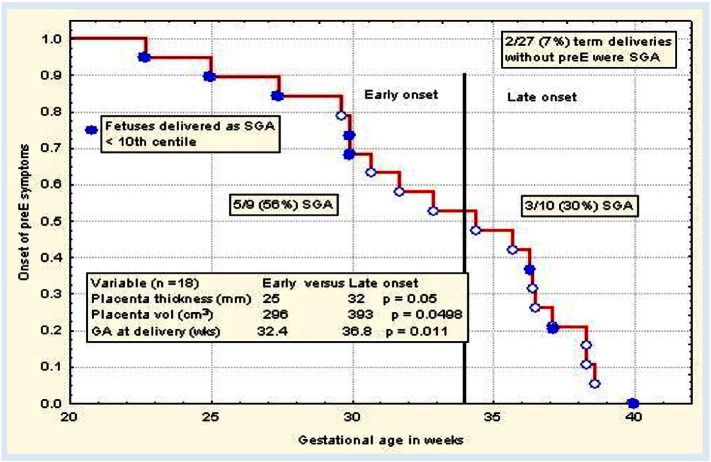
preE subjects are divided into early preE (before 34 weeks) and late preE (after 34 weeks) groups and compared their outcomes. The placental thickness in early preE subjects was 25 mm compared to 32 mm in late preE (p = 0.05) and placental volume in early preE 296 cm^3^ compared to 393 cm^3^ (p = 0.0498). Gestational age at delivery in early preE is 32.4 weeks vs 36.8 weeks in late preE (p = 0.011). About 56% of the infants (5 out of 9) who are born to early preE are small for gestational age (SGA) and 30% of the infants (3 out of 10) who are born to late preE are SGA.

We divided the preE subjects into early (before 34 weeks) and late preE (after 34 weeks) groups and compared their outcomes. The placental thickness in early preE subjects was 25 mm compared to 32 mm in late preE (p = 0.05) and placental volume in early preE 296 cm^3^ compared to 393 cm^3^ (p = 0.0498). Gestational age at delivery in early preE is 32.4 weeks vs 36.8 weeks in late preE (p = 0.011). About 56% of the infants (5 out of 9) who are born to early preE are small for gestational age (SGA) and 30% of the infants (3 out of 10) who are born to late preE are SGA (Fig. 1). Qualitative demographic variables are shown in Table 2.

### 3.2 WB data for p38 MAPK, Bax, Bcl-2, caspase-9 and Cox-2 proteins

**Fig. 2:**
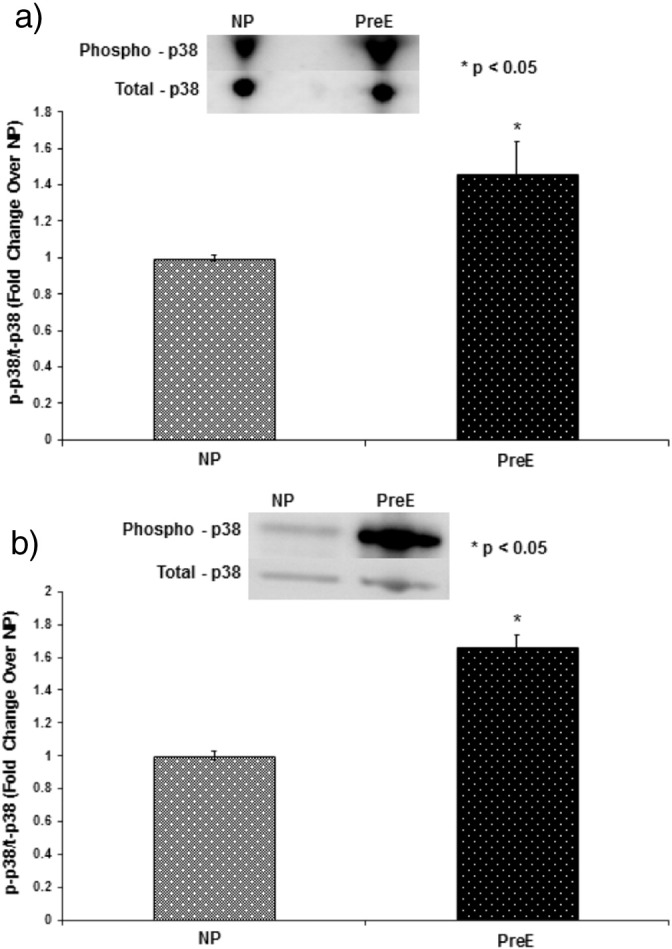
(a) The p38 MAPK phosphorylation was measured in the placental tissue from two groups of patients by Western blotting, running the homogenate from the placental tissues from NP and preE patients in gel followed by detecting with immunoblotting using anti-phospho-p38/anti-total-p38 antibodies. The patients were: normal pregnant (NP, n = 15) and preeclamptic (preE, n = 15). The placental p38 MAPK phosphorylation was significantly upregulated in preE patients compared to NP (*p < 0.05). The results presented are the mean ± SE. A blot from a representative experiment is shown in the figure. (b) The p38 MAPK phosphorylation was measured in the umbilical cord tissue from two groups of patients by Western blotting, running the homogenate from the cord tissues from NP and preE patients in gel followed by detecting with immunoblotting using anti-phospho-p38/anti-total-p38 antibodies. The patients were: normal pregnant (NP, n = 15) and preeclamptic (preE, n = 15). The cord p38 MAPK phosphorylation was significantly upregulated in preE patients compared to NP (*p < 0.05). The results presented are the mean ± SE. A blot from a representative experiment is shown in the figure.

**Fig. 3:**
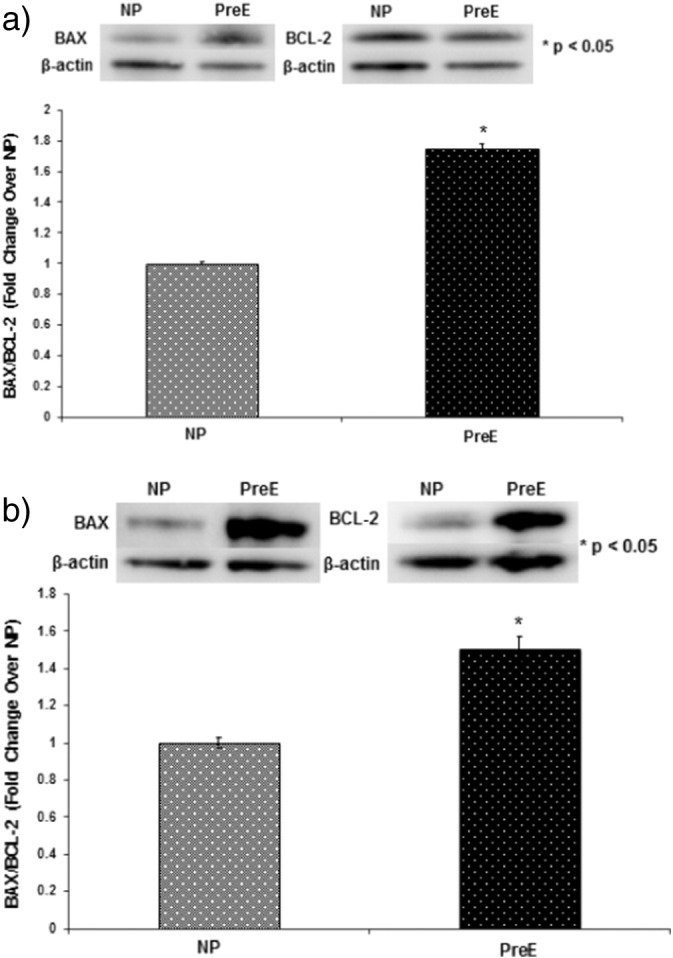
Representative blots of BAX/BCL-2 and beta-actin in placental (a) and umbilical cord (b) tissues from two groups of patients: NP (n = 15) and preE (n = 15). Graph presents means with SE of 15 experiments of each group for the expression of BAX/BCL-2 relative to beta-actin in the tissue lysates of (a) placental and (b) umbilical cord by Western Blot. The BAX/BCL-2 was significantly upregulated both in placenta and cord tissues of preE patients compared to NP (*p < 0.05). The results presented are the mean ± SE.

**Fig. 4:**
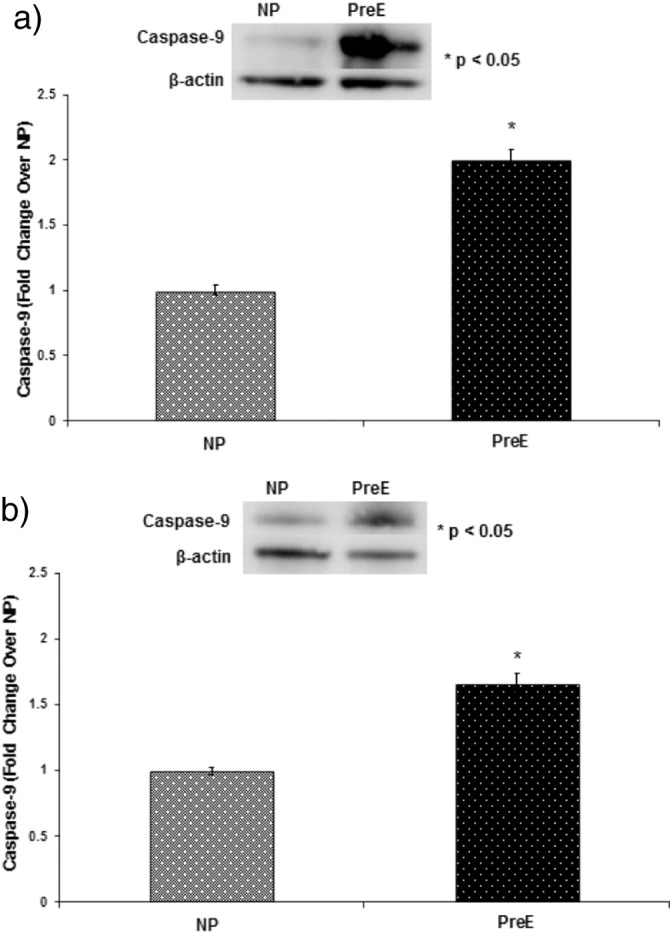
(a) Caspase-9 was measured in the placental tissue from NP and preE patients by immunoblotting using anti-caspase antibody. The patients were: normal pregnant (NP, n = 15) and preeclamptic (preE, n = 15). The placental caspase-9 was significantly upregulated in preE patients compared to NP (*p < 0.05). The results presented are the mean ± SE. A blot from a representative experiment is shown in the figure. (b) Caspase-9 was measured in the cord tissue from NP and preE patients by immunoblotting using anti-caspase-9 antibody. The patients were: normal pregnant (NP, n = 15) and preeclamptic (preE, n = 15). The cord caspase-9 was significantly upregulated in preE patients compared to NP (*p < 0.05). The results presented are the mean ± SE. A blot from a representative experiment is shown in the figure.

**Fig. 5:**
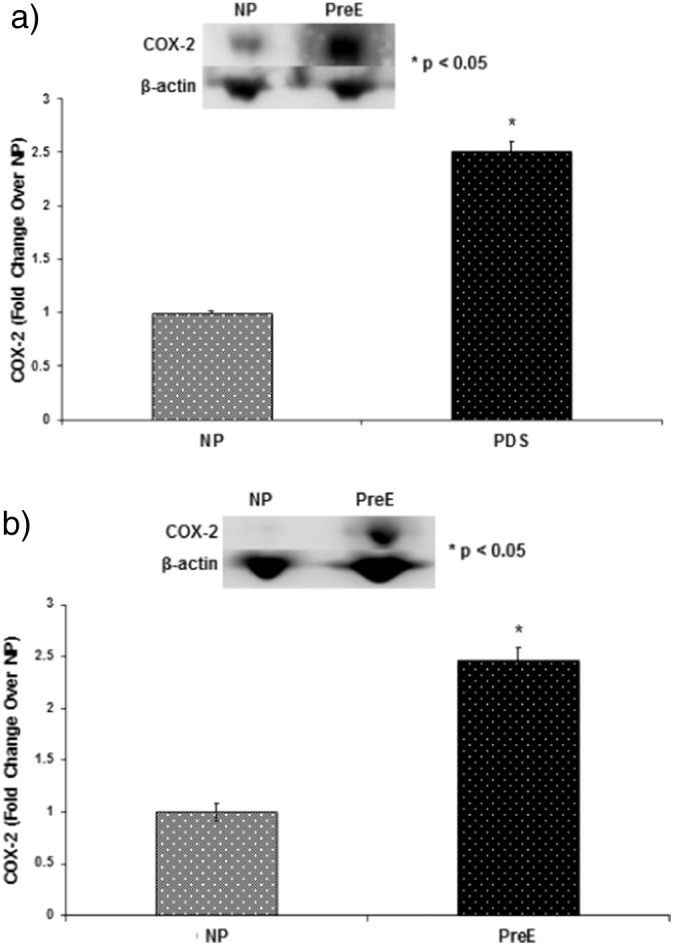
Representative blots of Cox-2 and beta-actin in placental (a) and umbilical cord (b) tissues from two groups of patients: NP (n = 15) and preE (n = 15). Graph presents means with SE of 15 experiments (each group) for the expression of Cox-2 relative to beta-actin in the tissue lysates of (A) placental and (B) umbilical cord by immunoblotting using anti-Cox-2 antibody. Cox-2 was significantly upregulated both in placenta and cord tissues of preE patients compared to NP (*p < 0.05). The results presented are the mean ± SE.

The p38 MAPK phosphorylation in both the placenta and umbilical cord was upregulated in preE patients compared to control (*p < 0.05) (Fig. 2 a–b). The expression of Bax/Bcl was higher in preE placenta and cord compared to normal (*p < 0.05 (Fig. 3 a–b). The expression of apoptotic signaling protein caspase-9 was higher in preE placenta and umbilical cord compared to NP (Fig. 4a–b). The Cox-2 expression was upregulated in placental and cord samples of preE patients compared to control (*p < 0.05 (Fig. 5a–b). We followed up the babies for both groups of patients after delivery for two weeks to assess the immediate pregnancy outcome.

### 3.3 Pregnancy outcomes

preE babies were stayed significantly longer period of time in hospital compared to NP babies (~ 3 vs ~ 26 days) after delivery for treatment of multiple complications. There were no complications in 6 infants, who are born to preE mothers. The complications of preE babies were as follows: 7 had hypoglycemia; 1 had hyperbilirubinemia; 7 had respiratory distress syndrome; 2 had bronchopulmonary dysplasia; 8 had intrauterine growth restriction; 6 had thrombocytopenia; 1 of each had retinopathy of prematurity (ROP), intraventricular hemorrhage (IVH) and hypoxic ischemic encephalopathy (HIE). However, so far there were no complications observed among the NP babies.

## 4 Discussion

The apoptotic and stress signaling proteins were significantly up-regulated in preE placentas and umbilical cords compared to normal pregnancies. The pregnancy outcomes for the preE pregnancies were complicated compared to normal pregnancies. Reduced blood supply leading to hypoxia from reduced uterine perfusion (RUPP) appears to be a significant pathophysiologic mechanism leading to preE. The feto-placental unit reacts by producing a cascade of inflammatory mediators and anti-angiogenic factors [20], [21]. The proposed mechanisms of reduced uterine perfusion and hypoxia, elevated antiangiogenic factors and systemic endothelial dysfunction are the significant pathophysiologic mechanism leading to the development of preE, and the higher BPs lead to increased stroke and atherogenesis [4], [20], [21]. RUPP seems to be one of the key determinants leading to hypoxia. The feto-placenta unit reacts by producing reactive oxygen species and cytokines, inducing a cascade of inflammatory mediators and anti-angiogenic factors [4], [20], [21]. This theory has been replicated in animal models. The timing of fetal exposure to preE is critical. Studies have demonstrated an increase in BP in RUPP animals compared with controls, whereas exposure to the effects of increased BP in late gestation indicate no difference in BP in the offspring compared to controls [22]. Placental hypoxia has been suggested as a contributing factor for the development of preE, in animal studies that surgically induced uteroplacental ischemia causes hypertension, proteinuria, and elevated levels of circulating sFlt-1 [23]. Hypoxic conditions have actually shown an increased invasiveness of CTBs, indicating that the primary malfunction must be abnormal placentation resulting in placental hypoxia [12], [13], [15].

The explanation of the pathogenetic mechanism of how preE causes the many deleterious effects in offspring has been studied extensively. A comprehensive analysis by Davis et al. showed that there is a 2.4 mm Hg increase in systolic BP in childhood and young adult life which is associated with an 8% increased risk of mortality from ischemic heart disease and a 12% increase risk from stroke [24], [25]. Preterm infants born to preE mothers also had lower flow-mediated dilatation which is a marker of endothelial dysfunction [26]. Endothelial dysfunction is an important indicator for subsequent cardiovascular disease and is present in preE [25], [27]. preE infants had higher BP and thicker carotid intima as a young adult. These can either be due to genetic predisposition or in utero exposure to inflammation, represented by the increased number of inflammatory markers in offspring of preE mothers [28]. preE mothers were found to have increased circulating levels of inflammatory markers, which correlates to the development of the syndrome [5], [26].

Shaker and Sadik [29] shown that increased oxidative stress in preE leads to lipid peroxidation results in elevation of Malondialdehyde (MDA, a marker of lipid peroxidation). They have also demonstrated increased caspase-9 activity and DNA fragmentation in preE secondary to increased oxidative stress. Zhang et al. [30] have reported AP-2α dependent regulation of Bcl-2/Bax modulates the apoptosis in trophoblast cells. They have shown that there is increased apoptosis in trophoblast cells in preE secondary to overexpression of AP-2α leading to imbalance between anti-apoptotic Bcl-2 and pro-apoptotic Bax signaling molecules resulting in preE. Mudgett et al. [31] have demonstrated the role of p38 MAPK in placental, embryonic and yolk sac angiogenesis. They have hypothesized that activation of p38 MAPK is required for the angiogenic response to the relative hypoxic state during early placentation. Webster et al. [32] have found that increased nitration of p38 MAPK decreases p38 MAPK activity resulting in poor placentation and preE development. Goksu Erol et al. [33] have demonstrated the roles of decreased iNOS expression and increased Cox-2 expression in decreased placental blood flow and increased resistance to the flow in the feto-maternal circulation resulting preE. Cawyer et al. have proven the hyperglycemia induced cytotrophoblast dysfunction and increased expression of p38 MAPK phosphorylation, PPARγ, Bcl-2-associated-X protein (Bax) and anti-apoptotic Bcl-2 ratio, caspase-9, and cyclooxygenase-2 (Cox-2) [34]. All of the above mentioned investigators had worked on different stress and apoptotic signaling molecules individually. In our experiment, we demonstrated expression of multiple signaling molecules like p38 MAPK, Bcl-2/Bax, caspase-9 and Cox-2 in preE and normal placental and cord tissues explaining their role in pathogenesis of preE.

preE is associated with endothelial dysfunction in the mother, which is related to the release of circulating vasculotoxic factors and the induction of augmented oxidative stress by the diseased placenta [6], [22], [25], [27], [28], [35]. These circulating factors may pass the placental barrier and leave persistent defects in the circulation of the offspring that may predispose to a pathological response later in life. Jayet et al. showed some evidence of vascular dysfunction in young offspring of mothers with preE [35]. It was found that pulmonary artery pressure is roughly 30% higher in offspring of mothers with preE than control subjects [35]. They also noted that brachial artery flow mediated dilatation is 30% lower in offspring of mothers with preE. The vascular dysfunction was related to preE itself because siblings of offspring of mothers with preE who were born after a normal pregnancy had normal vascular function [35].

According to a study done by Hakim et al., the rate of very early preterm delivery (less than 32 weeks of pregnancy) was 21.2% in uncomplicated preE and 37.2% in preE complicated by with prior chronic hypertension [22], illustrating the detrimental effect of hypertension alone on gestational age. Established preE serves as the basis for 15% of all preterm births [22]. Diseases of prematurity affecting every vital organ system have a higher frequency in the offspring of preE mothers due to the increased incidence of prematurity in this cohort. Neurologically, intraventricular hemorrhage and retinopathy of prematurity occur soon after birth more frequently in babies born to preE mothers; intellectual disabilities, hearing disabilities, and cerebral palsy plague these infants long-term [36]. Respiratory issues such as prolonged ventilator-dependence and bronchopulmonary dysplasia tied to developmental immaturity have been shown to occur more often in these infants in prospective studies [25]. Necrotizing enterocolitis, the most common gastrointestinal emergency of infants, occurs more frequently in this group in preE offspring [25], [37], [38].

The increased apoptotic signaling in preE which may lead to reduced nutrient transport capacity triggering placental release of vascular factors that produce maternal vascular responses characteristic of this syndrome. preE modifies the intrauterine environment by altering the pattern of hormonal signals and activating the detrimental cellular signaling that has been transported to the fetus. The fetus has to adapt to the change in intrauterine milieu and this adaptation increases the risk of disease to the offspring. Further prospective and retrospective studies with human subjects are ongoing by our research group to understand the mechanism of the transmission of detrimental signaling from mothers to the offspring. The stress and detrimental cellular signaling molecules that have been overexpressed in the placental and cord tissues from preE patients raises the possibility that those signals could be therapeutically blocked one day.

## Declaration of interest

The authors report no conflict of interest.

## Funding

Funding for this work was provided by Scott, Sherwood and Brindley Foundation and Department of Obstetrics and Gynecology (MNU) and the Noble Centennial Endowment for Research in Obstetrics and Gynecology (TJK).

## Transparency document

Transparency document. Image 1

## Figures and Tables

**Table 1:** Quantitative demographic variables (means ± SD (n)).

Variables	Patients with preeclampsia	Normal pregnancies	p-Value
Parity	2.1 ± 2.4 (20)	2.4 ± 1.5 (27)	0.11^b^
Maternal age (yrs) at delivery	28.8 ± 6.4 (20)	28.2 ± 4.4 (27)	0.74^a^
Maternal weight (pounds)	204 ± 50 (20)	177 ± 44 (27)	0.046^b^
Maternal height (inches)	65 ± 5 (20)	64 ± 3 (27)	0.69^b^
Maternal BMI (kg/m^2^)	34.2 ± 7.8 (20)	30.6 ± 6.9 (27)	0.095^a^
Systolic blood pressure (mm Hg)	166 ± 11 (20)	122 ± 10 (27)	< 0.0001^a^
Diastolic blood pressure (mm Hg)	93 ± 10 (20)	74 ± 9 (27)	< 0.0001^a^
Urinary protein (mg/24 h)	1974 ± 1149(20)	105 ± 17 (27)	< 0.0001^b^
Gestational age at delivery (weeks)	34.8 ± 4.0 (20)	39.2 ± 0.3 (27)	< 0.0001^b^
Baby weight (grams)	2287 ± 872 (20)	3528 ± 354	< 0.0001^b^
Rohrer's ponderal index	2.46 ± 0.32 (20)	2.94 ± 0.25 (27)	< 0.0001^a^
Infant length of stay (days)	20 ± 26 (20)	2 ± 0.5 (27)	< 0.0001^b^

a Comparison using Student's *t*-test.

b Comparison using Mann-Whitney *U* test.

**Table 2:** Qualitative demographic variables (proportions (%)).

Variables	Patients with preeclampsia	Normal pregnancies	p-Value^a^
Mode of delivery			
Cesarean delivery/total	12/20 (60%)^b^	24/27 (89%)^b^	0.021
Baby gender			
Male/total	8/20 (40%)	12/27 (44%)	0.76
Infant complications			
Yes/total	14/20 (70%)^c^	4/27 (15%)^c^	0.001
Infant size at delivered less than 10th percentile for gestational age (IUGR)			
Yes/total	8/20 (40%)	2/27 (7%)	0.007
Required NICU care			
Yes/total	13/20 (65%)	2/27 (7%)	< 0.0001

a Comparison using chi-square test.

b Need to list indications for cesarean sections for both groups with N for each indication.

c Complications for infants of the two study groups.
